# Demonstration of Anti-ambipolar Switch and Its Applications for Extremely Low Power Ternary Logic Circuits

**DOI:** 10.1021/acsnano.2c03523

**Published:** 2022-06-28

**Authors:** Yongsu Lee, Sunmean Kim, Ho-In Lee, Seung-Mo Kim, So-Young Kim, Kiyung Kim, Heejin Kwon, Hae-Won Lee, Hyeon Jun Hwang, Seokhyeong Kang, Byoung Hun Lee

**Affiliations:** Center for Semiconductor Technology Convergence, Department of Electrical Engineering, Pohang University of Science and Technology, Cheongam-ro 77, Nam-gu, Pohang, Gyeongbuk 37673, Republic of Korea

**Keywords:** anti-ambipolar, heterojunction, low thermal budget, ternary logic, pull-middle network, ternary full adder, low-power system

## Abstract

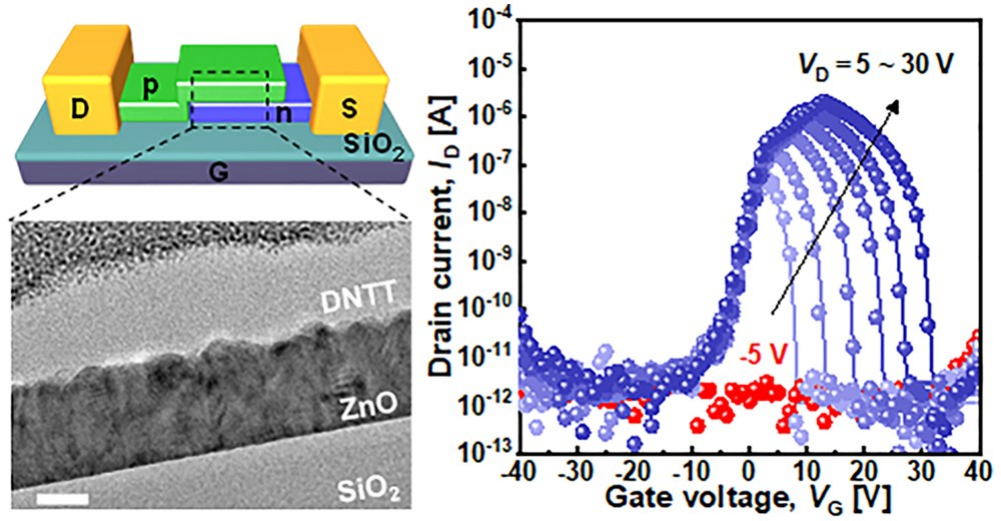

Anti-ambipolar switch (AAS) devices at a narrow bias region are necessary to solve the intrinsic leakage current problem of ternary logic circuits. In this study, an AAS device with a very high peak-to-valley ratio (∼10^6^) and adjustable operating range characteristics was successfully demonstrated using a ZnO and dinaphtho[2,3-*b*:2′,3′-*f*]thieno[3,2-*b*]thiophene heterojunction structure. The entire device integration was completed at a low thermal budget of less than 200 °C, which makes this AAS device compatible with monolithic 3D integration. A 1-trit ternary full adder designed with this AAS device exhibits excellent power–delay product performance (∼122 aJ) with extremely low power (∼0.15 μW, 7 times lower than the reference circuit) and lower device count than those of other ternary device candidates.

An increase in computing capabilities and integration density of CMOS devices has been achieved by reducing the minimum feature size of transistors.^1−5^ However, as the power consumption at the interconnect region now accounts for more than 90% of active power consumption for highly scaled chips, a progressive improvement in transistor structure alone cannot be a proper solution for the significantly increasing power consumption.^3,4,6−9^ Therefore, a more aggressive change in the chip architecture is necessary to deal with the power crisis for future data processing and other computing needs.

Multivalued logic (MVL) technology has been investigated as an alternative technology to overcome the significant rise in power consumption.^10−14^ MVL is a computing architecture operating with more than two logic states. Therefore, MVL provides significant benefits in terms of the number of transistors and interconnection length required to accomplish identical functions compared to binary logic. Especially, ternary logic, which consists of three logic states (0, 1, and 2 or −1, 0, and +1), is the most extensively studied among the MVL architectures because it theoretically provides the lowest power consumption and best noise margin.^15−20^

The major bottleneck in ternary logic is the absence of a unit ternary device that can be integrated with the semiconductor process and operated in the same manner as MOSFETs, *e*.*g*., room-temperature operation and high drive current. Thus, early studies on ternary architecture were performed using a combination of binary devices. The carbon nanotube field-effect transistor (CNTFET) has been widely used for ternary circuit research owing to its high mobility, tunable threshold voltage (*V*_th_), and very small physical dimensions. However, because of the difficulty in arranging multiple types of CNTs in an integrated circuit, studies on CNTFETs have only been performed theoretically.^17−19^ The graphene barristor, the Schottky barrier triode between graphene and a semiconductor, was also suggested as a unit transistor for ternary circuits by Heo *etal*. because of the easy integration process and tunability of *V*_th_ to control the Schottky barrier height.^21^

In addition to the combination of binary devices, ternary circuit implementation using a unit ternary device has been reported. For quantum dot gate FETs,^20^ an intermediate state was obtained by dynamic *V*_th_ shift owing to the tunneling charges accumulated in the quantum dot gate through a thin gate dielectric. However, it was difficult to fabricate two layers of well-aligned quantum dots, and the size limitation of quantum dots and the space between them restricted further scaling.

Recently, ternary devices with a dual-channel structure using graphene barristors have been suggested. The dual-channel ternary device utilizes two parallel channels with different *V*_th_ to obtain stepwise transfer characteristics. Kim *etal*. and Lee *etal*. demonstrated n-type and p-type dual-channel ternary devices with ZnO and dinaphtho[2,3-*b*:2′,3′-*f*]thieno[3,2-*b*]thiophene (DNTT), respectively, and complementary ternary circuits were demonstrated.^22,23^ Furthermore, ternary transistors with a stack-channel structure having two stacked ultrathin semiconductor channel layers with different *V*_th_ have been demonstrated.^24,25^ In this device, the intermediate state was realized using an ultrathin phase composite ZnO layer having a voltage-independent constant current flow mechanism, which is attributed to the finite density of states in the conduction band from the mobility edge quantization phenomenon. Low process temperature and easy fabrication process make this device compatible with monolithic 3D integration.

A major challenge in ternary circuit design is finding a proper method to handle the intermediate state. When both devices in the ternary inverter are simultaneously in the intermediate state, a leakage current path between *V*_DD_ and *V*_GND_ is formed, leading to intrinsic power dissipation.^17−25^ This leakage current mitigates the benefits of ternary logic, which is primarily associated with lower device count and less power consumption.

To avoid this problem, a ternary circuit scheme, called single pole triple throw (SPTT), has been proposed.^26^ It uses an additional power rail for *V*_DD_/2. Although it becomes much more straightforward to obtain three states with this approach, the number of devices to form three states inevitably increases. Usually, two binary switches represent states 0 and 2, and two additional devices are necessary to represent state 1. The addition of power rails was technologically challenging in the 1970s; however, the recent progress in buried power rail technology can alleviate the area burden of additional power rails and contacts.^27,28^ Thus, the SPTT approach became a promising technology for ternary architecture.

A single anti-ambipolar switch (AAS) device can replace the two devices required to emulate the intermediate state of a standard ternary inverter (STI) designed with an SPTT scheme. AAS devices pass information at a sharply limited gate bias range corresponding to the intermediate state, *V*_DD_/2. Thus, an appropriately designed AAS device can be used as a unit device in a pull-middle (PM) network, which is connected with a *V*_DD_/2 power rail. Peak-to-valley ratio (PVR), defined as the ratio of on (peak) current and off (valley) current, is one of the important characteristics of AAS devices. To implement the proposed ternary circuit, a high PVR is desirable because a high on current makes state transition fast, and a low off current reduces the power consumption due to the leakage current between networks, similar to the circuits using unipolar devices.

Several prior studies on AAS behavior used bias-dependent modulation of transconductance (*g*_m_) curves in Si, Ge, and III–V semiconductor devices.^29−35^ Unfortunately, the low PVR in these devices limited their practical applications. Recently, various heterojunction devices using transition metal dichalcogenides (TMDCs), such as MoS_2_, WSe_2_, SnSeS, and ReS_2_, were proposed, and high current density and moderate PVR have been reported.^36−40^ However, the difficulty of large-area integration of TMDC heterojunction devices and their compatibility with CMOS circuits are still a hurdle for practical applications. Thus, manufacturing an AAS device compatible with large-scale CMOS integration and having a reasonable performance at room temperature is one of the most important challenges in ternary logic technology.

Here, we report a ZnO–DNTT AAS device, with a high PVR of above 10^6^, adjustable operation bias range, solid stability and reliability, and CMOS process compatibility, especially with back-on-the-line or 3D integrated circuits. The performance of the ternary full adder designed with the ZnO–DNTT AAS device exhibited ∼7 times lower power–delay product (PDP) than the ideal CNTFET-based ternary circuits in testing.

## Results and Discussion

**Figure 1:**
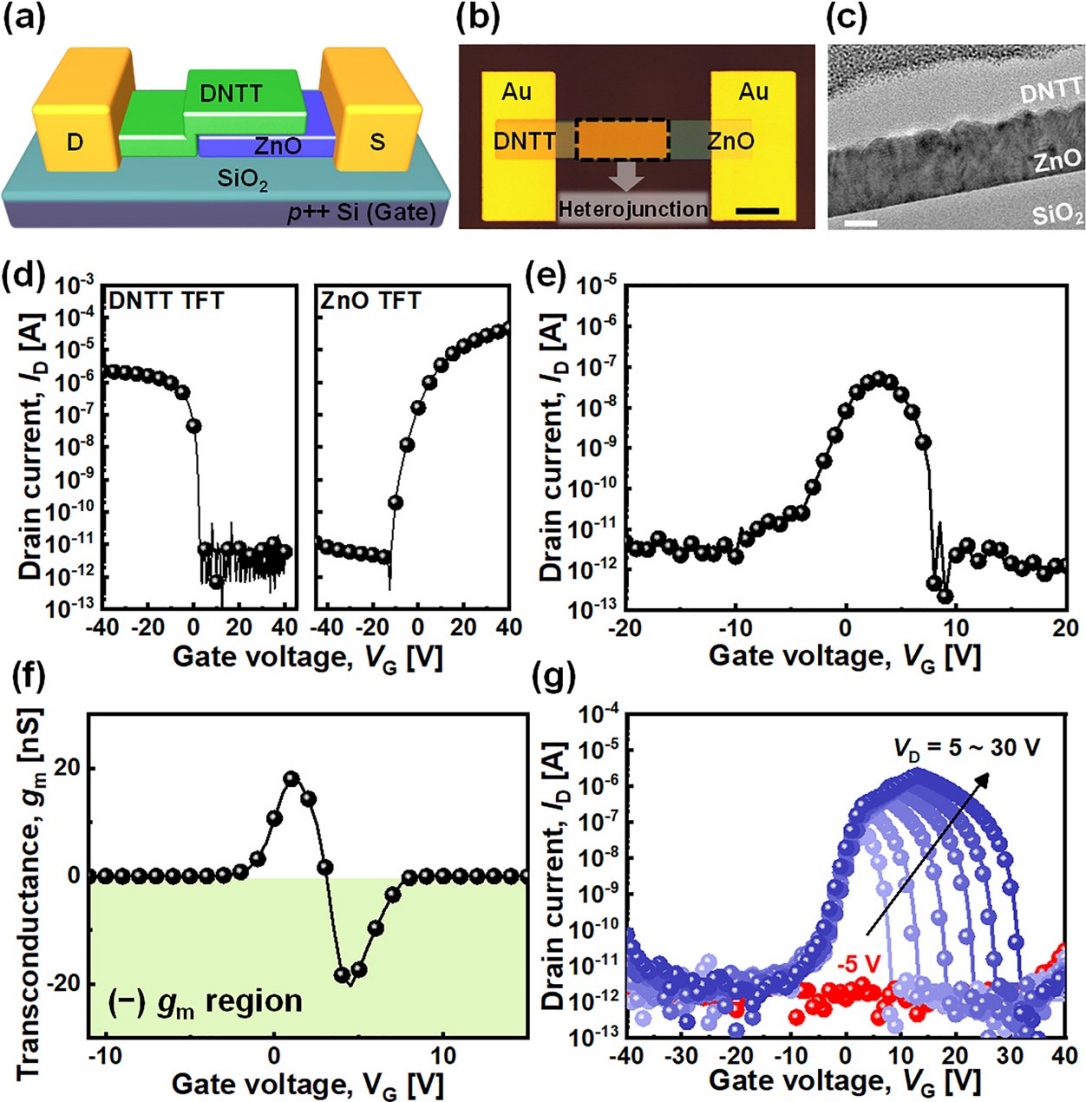
(a) Schematic device structure of the ZnO–DNTT AAS device. (b) Photograph of the fabricated device. Scale bar = 200 μm. (c) Cross-sectional TEM image of the ZnO–DNTT heterojunction region. Scale bar = 20 nm. (d) *I*_D_–*V*_G_ curves of p-type DNTT TFT and n-type ZnO TFT. The thicknesses of each channel are 50 and 29 nm at *V*_D_ = ∓ 5 V, respectively. (e) *I*_D_–*V*_G_ curves of ZnO–DNTT AAS devices at *V*_D_ = 5 V. (f) Negative *g*_m_ property of the AAS device at *V*_D_ = 5 V. (g) *I*_D_–*V*_G_ curves of ZnO–DNTT AAS devices with *V*_D_ ranging from −5 and 5–30 V. Symbols indicate experimental data, and lines indicate device modeling fitting results.

Figure 1(a) shows the schematic device structure of the ZnO–DNTT AAS device. Bulk p++ Si substrate and 90 nm SiO_2_ are used as back gate and gate dielectric, respectively. On top of the gate dielectric, ZnO and DNTT channels are formed in sequence. Both semiconductors were selected because of the low process temperature required for monolithic 3D integration and air-stable characteristics required for device integration. ZnO was stably deposited using a low-temperature ALD process at 120 °C.^41,42^ DNTT, a p-type organic thin-film semiconductor, was also deposited at room temperature using thermal evaporation. Both ZnO and DNTT showed reasonable air stability. The heterojunctions of the ZnO and DNTT layers partially overlap with each other, and Au electrodes contact each side of the heterojunction channels. The fabrication process is detailed in the Methods section (and Supporting Information, Figure S1). Figure 1(b) shows the fabricated device, with device dimensions of *W* = 180 μm and *L* = 420 μm for the overlapped regions and *L* = 300 μm for the underlapped regions. Figure 1(c) shows the cross-sectional TEM image of the ZnO–DNTT heterojunction. The XPS analysis of the ZnO channel indicates that ZnO is a bundle of nanorods having a wurtzite structure. For DNTT, a benzene ring structure with sulfur atoms conjugated with carbon atoms is verified by XPS analysis (Supporting Information, Figure S2).

Figure 1(d) shows the *I*_D_–*V*_G_ curves of thin-film transistors (TFTs) from individually fabricated DNTT and ZnO TFTs with typical electrical characteristics of depletion mode p-type and n-type semiconductor TFTs, respectively. The p-type DNTT TFT exhibited a subthreshold swing of 0.33 V/dec, a field-effect mobility of 2.3 cm^2^/V·s, and an on/off ratio of 10^6^. For the ZnO n-type TFT, a subthreshold swing of 1.12 V/dec, a field-effect mobility of 48 cm^2^/V·s, and an on/off ratio of 10^7^ were obtained. Swing values appear to be high in our devices because the gate dielectric thickness is 90 nm. The on/off ratio and field-effect mobility of our devices are comparable to or better than those of individual n-type ZnO TFT and p-type DNTT TFTs previously reported.^41−44^ Furthermore, owing to the balance of these electrical characteristics between n- and p-type TFTs, the AAS devices fabricated with the combination of ZnO TFT and DNTT TFT showed symmetrical transfer properties.

The transfer characteristic of ZnO–DNTT AAS devices measured at *V*_D_ = 5 V is shown in Figure 1(e). A PVR of ∼10^5^ was obtained within a narrow turn-on region from −3 to 8 V. The AAS device showed a negative *g*_m_, which reached −20 nS at *V*_DNTT_ = 5 V, as shown in Figure 1(f). Figure 1(g) shows the drain bias dependence of the AAS device. As *V*_D_ increased, the peak voltage, peak current, and operation window of the AAS device increased, and a PVR of ∼10^6^ was obtained at *V*_D_ = 30 V. This is the highest PVR of an AAS device yet reported (10^2^–10^5^).^29−40,45,46^ On the other hand, when *V*_DNTT_ is negative (−5 V), the current does not flow because of the reverse bias barrier of the PN junction.

**Figure 2:**
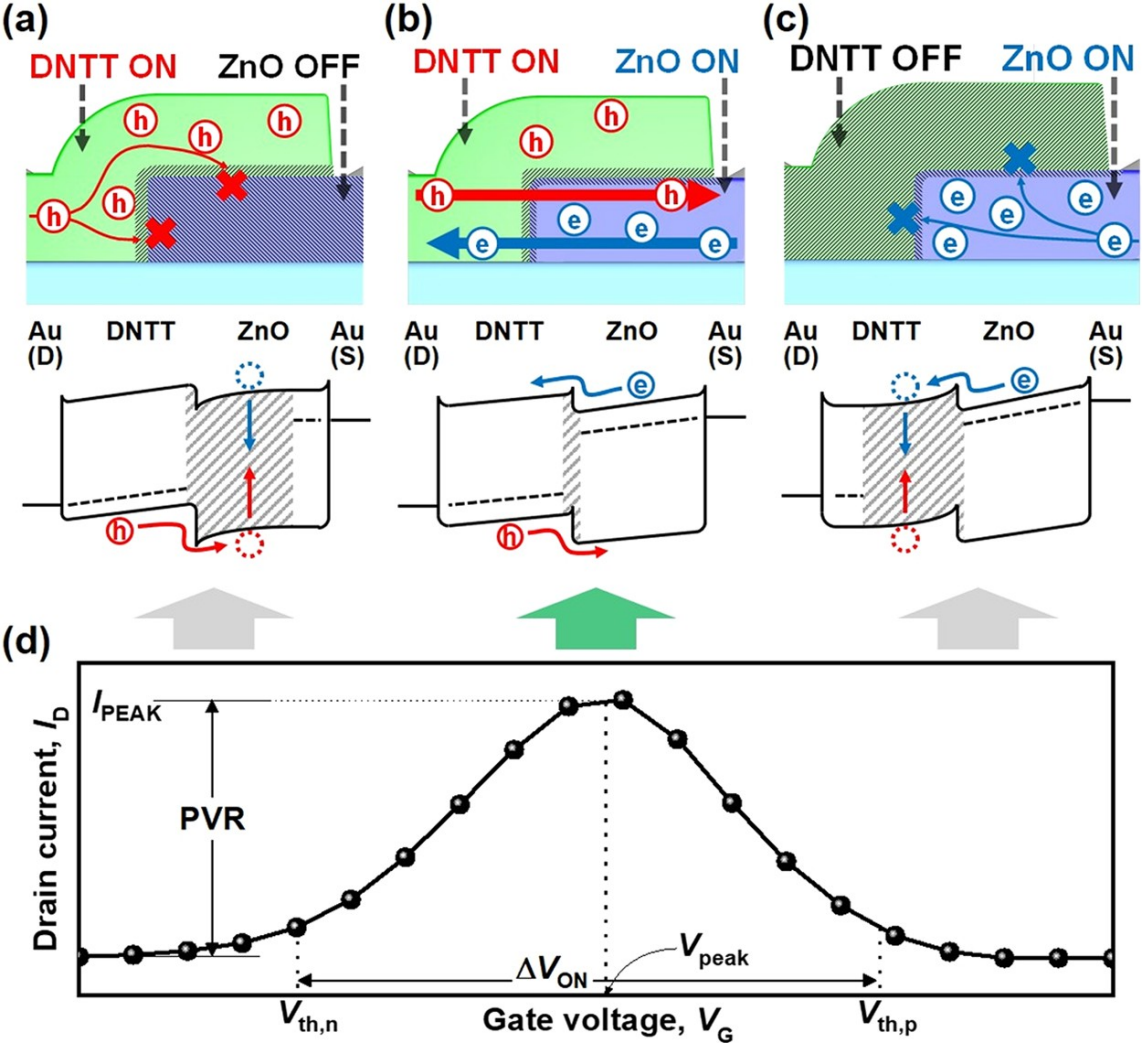
Schematic diagram of the cross-sectional AAS device structures and band diagrams with carrier flows when (a) *V*_G_ < *V*_th,n_, (b) *V*_th,n_ < *V*_G_ < *V*_th,p_, and (c) *V*_th,p_ < *V*_G_. (d) Typical *I*_D_–*V*_G_ curve of the AAS device.

Figure 2 shows the operation mechanism of the AAS device. The electrical behavior of the AAS device can be easily understood if we interpret this device as three devices connected in series that are controlled by the same gate bias: n-type ZnO TFT–ZnO/DNTT PN junction–p-type DNTT TFT. When *V*_G_ was lower than *V*_th_ of the ZnO TFT (*V*_th,n_) (Figure 2(a)) or higher than *V*_th_ of the DNTT TFT (*V*_th,p_) (Figure 2(c)), the final conductance of this series connected device was very low; that is, when one of two semiconductors is turned off, the carriers cannot flow across the depleted channel. Forward diode current can flow through the ZnO/DNTT heterojunction region only when the ZnO TFT and DNTT TFT regions are partially turned on. Thus, this device can only be turned on between *V*_th,n_ and *V*_th,p_ (Δ*V*_ON_) (Figure 2(b)). As a result, the current flow increases rapidly at a certain bias and dissipates at the other bias, as shown in Figure 2(d). *I*_PEAK_ position is defined as the highest current value at *V*_PEAK_, and the peak-to-valley ratio is defined as *I*_PEAK_ over the lowest current outside of Δ*V*_ON_.

**Figure 3:**
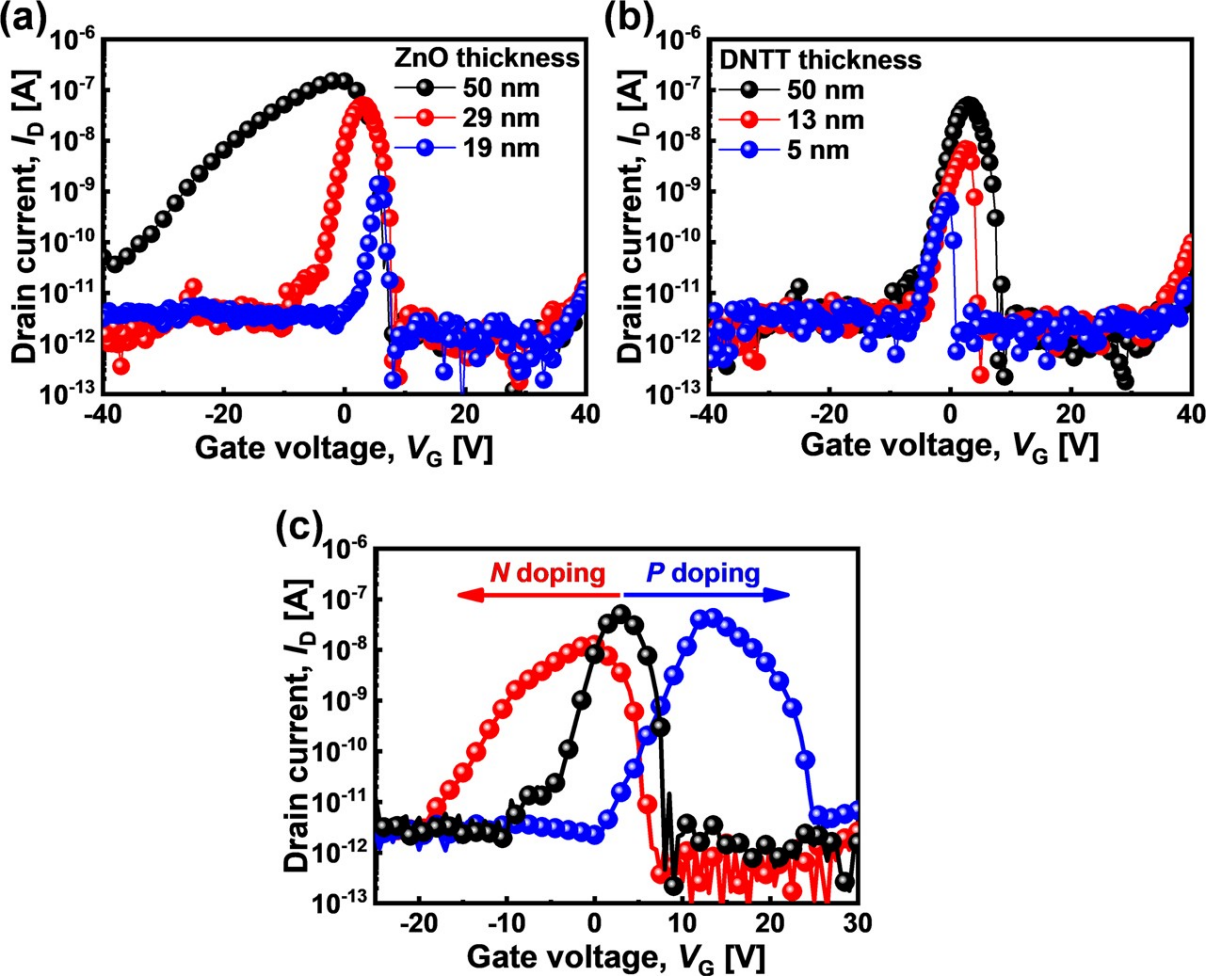
*I*_D_–*V*_G_ curves of the AAS device with different (a) thicknesses of ZnO (50 nm of DNTT), (b) thickness of DNTT (29 nm of ZnO), and (c) types of doping layer at *V*_D_ = 5 V (29 and 50 nm of ZnO and DNTT, respectively).

Physically, the drive current of this device is similar to the leakage current of an inverter consisting of a p-type DNTT TFT and an n-type ZnO TFT, but the current flux can be modulated by individually controlling the properties of an n-type TFT, a p-type TFT, and ZnO/DNTT heterojunctions. Figure 3(a) shows the outcome of device modulation with different ZnO channel thicknesses. As the ZnO channel thickness increases, the electron charge concentration tends to increase, so the *V*_th_ of ZnO TFTs decreases.^25,47^ Accordingly, the turn-on range (Δ*V*_ON_) of the AAS device can be modulated. By controlling the thickness of ZnO, *I*_PEAK_ was modulated in proportion to Δ*V*_ON_. In the DNTT channel, the hole charge concentration can also be varied by changing the thickness of the channels.^48^Figure 3(b) shows the *I*_D_–*V*_G_ curves of AAS devices with different DNTT channel thicknesses. Because the *V*_th_ variability of individual ZnO TFTs was much larger than that of DNTT TFTs (Supporting Information, Figure S4(a–b)), the range of Δ*V*_ON_ modulation was higher for devices with different ZnO thicknesses than for those with different DNTT thicknesses. Meanwhile, even though there was a trade-off between *I*_PEAK_ and Δ*V*_ON_, very sharp and narrow regions of Δ*V*_ON_ could be achieved with extremely high PVRs above 10^3^ in both cases.

While the thickness change in the n-type or p-type region affected the *V*_th_ and current level, the peak current position was not changed significantly. In other words, shifting the range of the turn-on region of the device was limited. To find a way to shift the device turn-on region, an additional polymer-based doping layer was inserted between the ZnO and DNTT layers. Figure 3(c) shows the result of the doping layer insertion. We used polyethylenimine (PEI) (n-doping) and poly(acrylic acid) (PAA) (p-doping) as doping layers, which induced electrons and holes at both surfaces of ZnO and DNTT, respectively. With electron doping (PEI), the *V*_th_ of single-channel TFT devices shifts toward the negative bias side. Meanwhile, the *V*_th_ shifts toward the positive bias side with hole doping (PAA) (Supporting Information, Figure S4(c–d)). Figure 3(c) clearly shows that the range of the turn-on region of the device can be shifted by inserting the doping layer. In summary, we have identified various parameters that can be used to modulate the peak current level, peak position, and width of the turn-on region. With further optimization, we expect to realize a more sharply defined turn-on region at the specific design position.

**Figure 4:**
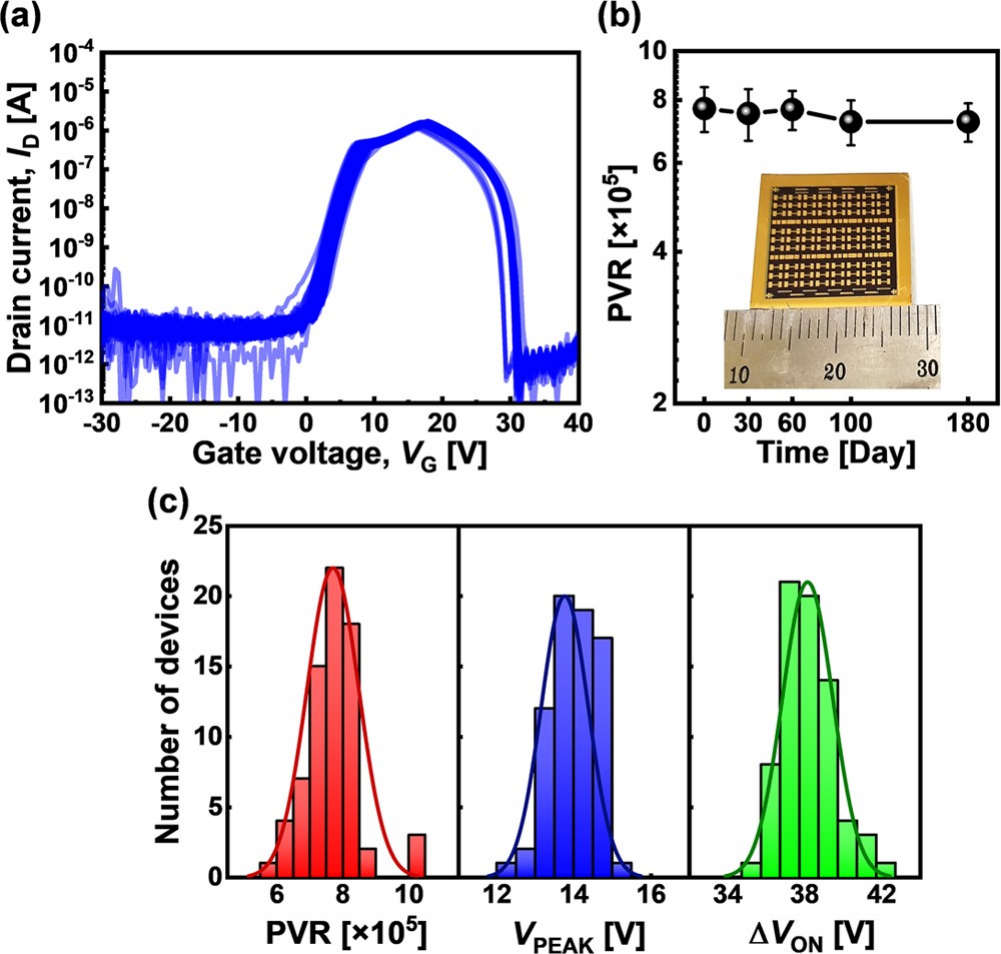
(a) *I*_D_–*V*_G_ curves for 72 separate AAS devices at *V*_D_ = 30 V, in which thicknesses of ZnO and DNTT are 29 and 50 nm, respectively. (b) PVR of the AAS device over time (inset: 2 cm × 2 cm wafer with 72 AAS devices) and (c) the distributions of the PVR, *V*_PEAK_, and Δ*V*_ON_ from 72 AAS devices.

The fabrication process appears to be relatively stable and shows promising scale-up feasibility. Figure 4(a) shows consistent *I*_D_–*V*_G_ curves for 72 separate AAS devices in a single chip. To verify the stability and manufacturability of ZnO–DNTT AAS devices, the statistical data were extracted. Figure 4(b) shows the average PVR values measured over 180 days, having a consistent distribution within a 2 × 2 cm chip (inset of Figure 4(b) shows the optical photograph of this chip). Figure 4(c) shows the distributions of PVR values, *V*_PEAK_, and Δ*V*_ON_. The coefficient of variation, CV = *s*/*x*_m_ × 100%, where *s* is the standard deviation and *x*_m_ is the average value, can be applied to quantify the distribution of data.^49^ The CVs of the distributions for PVR, *V*_PEAK_, and Δ*V*_ON_ are 9.7%, 4.4%, and 3.4%, respectively. CVs of each distribution are within 10% for all devices measured from the same chip, which means the device distributions are reasonably tight even with the manufacturing process at university facilities (clean class 10000). Table 1 compares various AAS devices reported in the literature. Most of the devices fabricated with TMDCs used the exfoliation method, which is not suitable for practical large-area manufacturing processes. Some heterojunction devices were fabricated using organic semiconductors deposited using the evaporation method, but the operation voltages were very high.^45,46^ Our ZnO–DNTT AAS device exhibited air-stable and uniform device properties with a reasonably tight distribution and can be fabricated with a low process temperature, which is very useful for monolithic or heterogeneous 3D integration.

**Table 1:** Comparison of AAS Devices

device	*J*_D_ [nA/μm]	PVR	fab. method	fab. *T* [°C]	EOT [nm]	|*V*_D_| [V]
ZnO–DNTT (this work)	0.9–5.6	10^5^–10^6^	ALD and evaporation	120	90	5–30
Si (p^+^–i–n^+^)^29^	0.004–2	2–10	CMOS		25	0.05–0.7
Si (n^+^–p^+^–n^+^)^30^	3.6	2	CMOS		2	0.001
Si (p^+^–n^+^–p^+^)^31^	20–109	1–5.5	CMOS	800	3	0.01–0.05
Si–Ge nanowire^32^	0.22–270	20–48	CVD	495	2	0.2–0.8
GaSb–InAsSb nanowire^33^	2200	1.6	epitaxy	500	10	0.5
MoS_2_–WSe_2_^36^	30	10^3^	exfoliation	360	>300	1
SnSeS–BP^37^	133	10^2^	exfoliation		4.68	1
MoS_2_–BP^38^	67	10^3^	exfoliation	250	300	1
ReS_2_–BP^39^	4000	10^5^	exfoliation	250	3.9	1
MoS_2_–pentacene^40^	450	10^3^	exfoliation	100	300	10
PTCDI-C8-6T^45^	0.2	5 × 10^4^	evaporation	120	>200	60
PTCDI-C8–DNTT^46^	0.05–0.6	10^3^–10^5^	evaporation	175	>350	30–60

For more practical applications, circuit-level implementation strategies of ternary circuits are examined using the ZnO–DNTT AAS device model. The semiphysical analytical device model is developed using a series connection of n- and p-type TFT models and a PN junction model. The simulation result fits very well with the experimental data shown in Figure 1(g).

**Figure 5:**
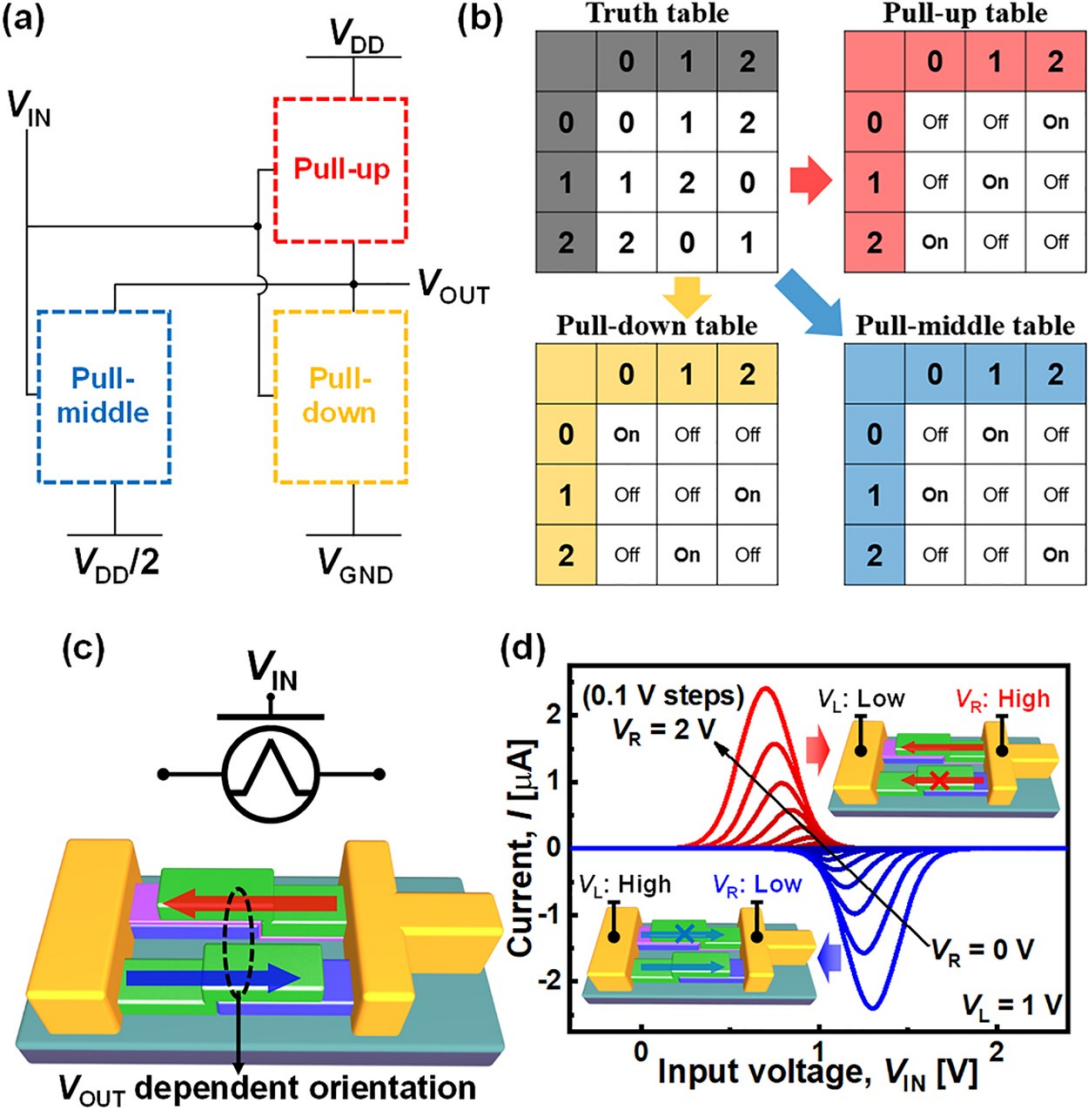
(a) Static gate design methodology for the proposed ternary circuit, (b) their truth table and switching table strategy, (c) device symbol and device structure of an AAS ternary unit cell, and (d) current transfer characteristics of an ideal AAS ternary unit cell model for EOT = 1 nm and *W* = *L* = 100 nm.

Conceptually, the ternary circuit using the AAS device can be simplified as shown in Figure 5(a). Three networks for pull-up (PU), pull-down (PD), and pull-middle (PM) are necessary to represent ternary states, and they are connected to *V*_DD_, *V*_GND_, and *V*_DD_/2 voltage sources, respectively. Compared with previous ternary circuit schemes (Supporting Information, Figure S5), an additional voltage source (*V*_DD_/2) and a PM network were added to provide an intermediate state. Recently, an inverter showing ternary characteristics using an organic-based AAS device has been reported.^46^ In this scheme, the leakage current problem at the intermediate state still exists because both PU and PD are half turned on. In our scheme, devices in PU and PD networks turn off at the intermediate state, which is similar to that in a CMOS inverter circuit. Within the narrow operation window where both PU and PD networks are turned off, the AAS device is turned on to provide the intermediate state while avoiding the leakage current from *V*_DD_ to *V*_GND_. In summary, three networks are used to represent three states in our scheme, and only one network is turned on for each state.

The static ternary gate design methodology using this approach is shown schematically in Figure 5(b). The truth table is divided into three tables, which correspond to PU, PD, and PM networks. Note that the AAS device works only under a forward drain bias (*V*_D_ = +5 V). When the drain bias is negative, there is no current flow, as shown in Figure 1(g). In this case, the transition between the PM and PU (or PD) network can be restricted if a reverse bias is applied to the AAS device during the transition. This problem can be overcome by adding an AAS device in the opposite direction as shown in Figure 5(c). Figure 5(d) shows the theoretically modeled *I*–*V* curves of an AAS unit cell, which shows bidirectional switching characteristics with *V*_DD_ less than 2 V. For this modeling, the equivalent oxide thickness (EOT) of the gate dielectric is assumed to be 1 nm, and the channel length and width are assumed to be 100 nm to emulate the scaled device operation.

**Figure 6:**
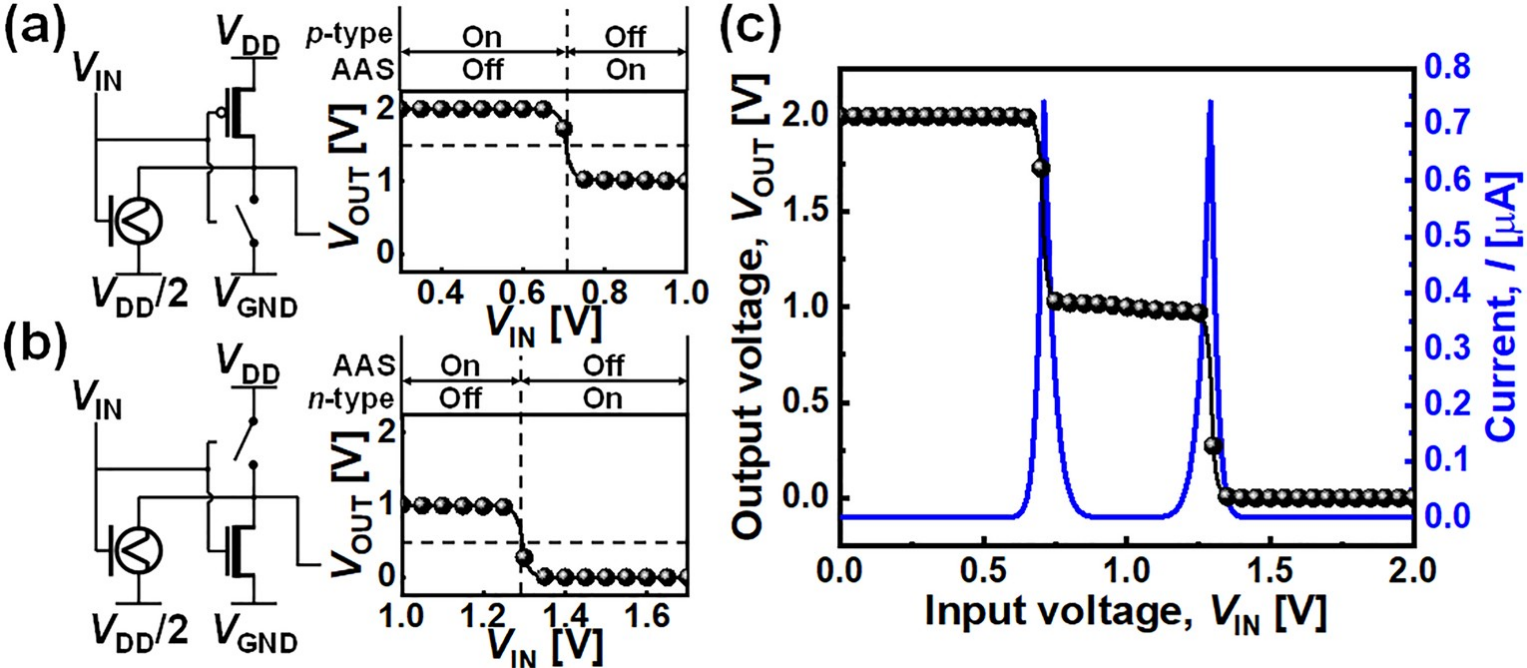
Proposed STI operation and its VTCs as *V*_OUT_ changes from (a) 2 to 1 V and (b) 1 to 0 V and (c) whole VTC (symbol) with drive current (line).

Using the AAS unit cell shown in Figure 5(c), an STI is designed to demonstrate the circuit level benefits of the ZnO–DNTT AAS device. Figure 6(a) shows the transition from a PU to PM network. With low *V*_IN_, the p-type device in the PU network is turned on and the AAS device in the PM network is turned off. As *V*_IN_ increases, the p-type device is starting to turn off while the AAS device is starting to turn on. When the transition between the PU and PM network occurs, *V*_OUT_ is changed from 2 V (*V*_DD_) to 1 V (*V*_DD_/2). The leakage current flows from the *V*_DD_ node to the *V*_DD_/2 node during this state transition only, similar to that in a CMOS inverter. Likewise, Figure 6(b) shows the transition between the PM and PD network when *V*_OUT_ is changed from 1 V (*V*_DD_/2) to 0 V (*V*_GND_). The voltage transfer characteristics (VTCs) and the leakage current are shown in Figure 6(c). In both cases, the leakage current flows only during the state transition, in which both networks (PU and PM or PM and PD) are turned on at the same time. As a result, the static power problem at the intermediate state of ternary logic can be minimized. In other STIs, leakage currents at the intermediate state are on the order of μA to nA,^19,25^ but the leakage current of the STI with an AAS device is at the pA level. Overall, the static power of an STI with an AAS device can be reduced to a sub-nW level, which is ∼1/100 of the typical value for a CNTFET-based ternary STI (Supporting Information, Figure S6).

**Figure 7:**
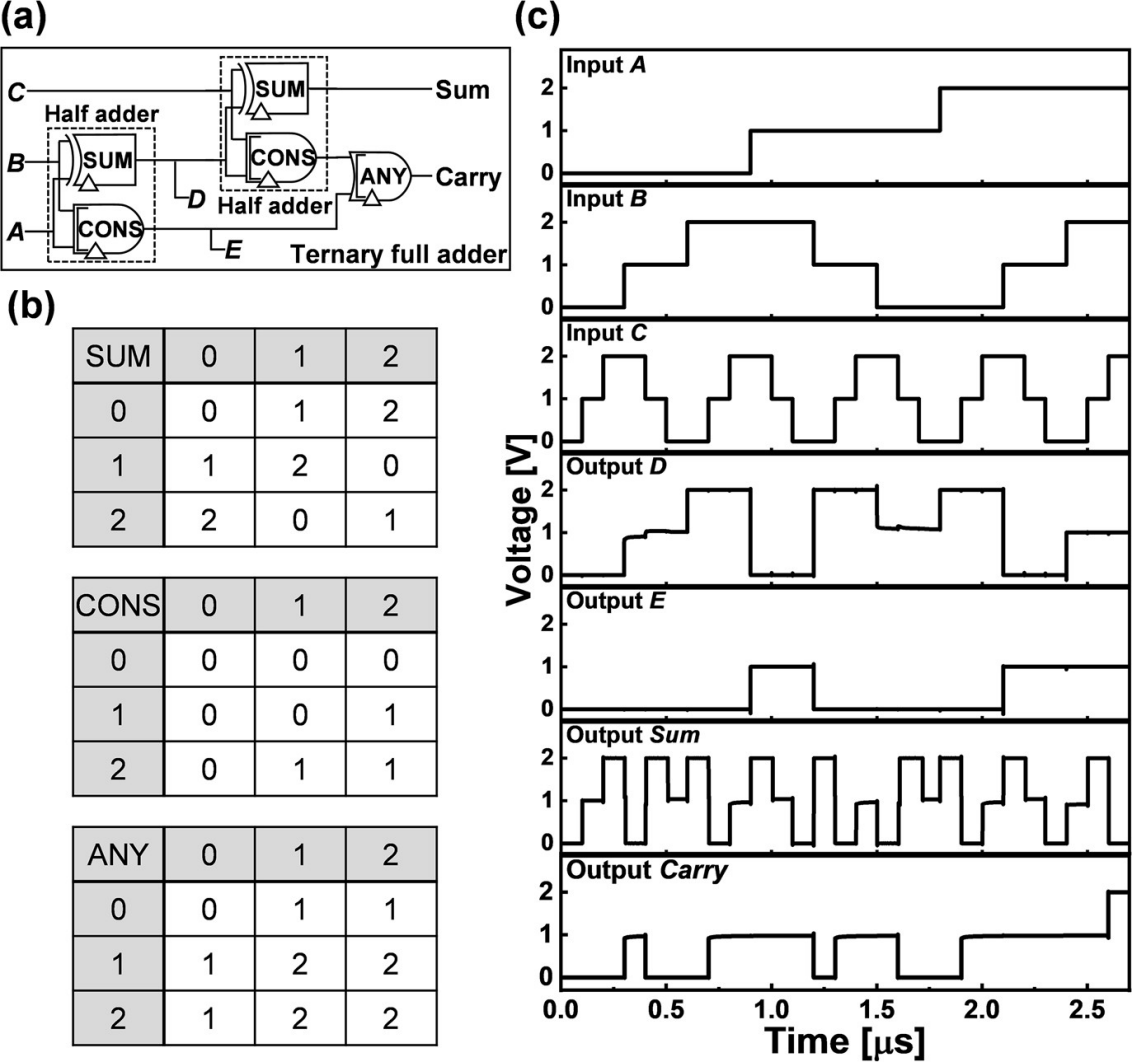
(a) Gate level of the ternary full adder, (b) truth tables of SUM, CONS, and ANY gates, and (c) transient responses of the proposed ternary full adder.

Using the above design strategy, various ternary module circuits such as STI, negative ternary inverter (NTI), positive ternary inverter (PTI) (Supporting Information, Figure S7), NMIN, NMAX (Supporting Information, Figure S8), SUM, CONS, and ANY (Supporting Information, Figure S9) were designed using the AAS devices. Integrating these module circuits, a ternary full adder was simulated. Figure 7(a) shows a ternary half adder designed by combining one SUM and one CONS gate. A ternary full adder is designed by combining two half adders and one ANY gate.^19,21^ The truth tables of SUM, CONS, and ANY gates are shown in Figure 7(b). The transient responses of the ternary full adder obtained at 5 MHz frequency are shown in Figure 7(c). Because these devices are not fully optimized, some glitches remain; however, the overall logic functionality has been successfully confirmed, which indicates that ternary logic circuits of any size can be designed using our approach.

The performance of our ternary full adder implemented with the ZnO–DNTT AAS device was evaluated and compared with other circuits having similar complexity using *HSpice* (Table 2). In all the circuits, four inverters were connected to the fan-out node, and all scaled physical dimensions were set to 100 nm (for binary CMOS, it was 90 nm). The CNTFET model of Stanford University^50^ and the predictive technology model for a 90 nm CMOS^51^ are used to evaluate other full adder circuits. For the binary ternary adder, 2-bit circuits are used for the comparison because the size of data of 1 trit is closest to that of 2 bits.^21,52^

**Table 2:** Comparison of Full Adder Circuits

logic architecture		*V*_DD_ [V]	# Tr	power [μW]	delay [ps]	PDP [aJ]
1-trit ternary	this work (*L = W* = 100 nm)	2	88	0.15	799	122
	CNTFET (*L* = 100 nm)	1	110	1.07	160	172
2-bits binary	90 nm CMOS	2	56	7.15	120	858
		1	56	0.096	200	19

Compared to the CNTFET-based 1-trit full adder, the proposed full adder has great advantages in terms of the number of transistors, power, and power–delay product, even at larger *V*_DD_. In particular, the power consumption is ∼7 times lower than that of the CNTFET-based full adder. Furthermore, the power consumption and PDP of the 1-trit full adder are superior to the 2-bit binary full adder; especially, the power consumption is ∼47 times lower than that of the binary full adder. The power consumption of the ternary circuit is even comparable to that of the optimal CMOS circuit at *V*_DD_ = 1 V. Although the PDP of the ternary circuit is comparable to that of binary CMOS due to significant power saving, the delay needs to be improved further, by potentially optimizing parasitic components such as contact resistances or capacitances. Overall, we have confirmed that the proposed ternary circuits utilizing the AAS device can have technical advantages for extremely low power applications if the appropriate scaling of the AAS device can be achieved.

## Conclusion

A ZnO–DNTT AAS device and ternary unit cell design to overcome the well-known leakage current problem of ternary logic in the intermediate state has been successfully demonstrated with extremely low power operation capability. Using this device concept, the advantages of the ternary full adder over other ternary devices or even binary CMOS devices, especially in terms of power consumption, have been confirmed. Even though the operation voltage of the AAS device should be scaled down further for more scaled devices, this approach will expand the capabilities of ternary logic architecture, especially for low-power ternary logic systems monolithically integrated with binary logic systems.

## Methods

### Fabrication Process and Electrical Characterization of ZnO–DNTT AAS Device

The 90 nm SiO_2_/p++ Si substrates (used as a back gate) were cleaned with acetone, isopropyl alcohol, and distilled water (DI) for 5 min in sequence by sonication. Then, ZnO layers were deposited using the atomic layer deposition (ALD) process at 120 °C using a diethyl zinc (DEZ) precursor and H_2_O oxidant. The thicknesses of ZnO varied from 19 to 50 nm by controlling the cycle of the ALD process. Then, the ZnO layers were patterned using photolithography and a wet etch process with 1% HCl solution diluted by DI. To perform chemical doping, the surface of active ZnO was submerged into a 0.2 wt % ethanol-diluted branched PEI (Sigma-Aldrich) or PAA (Sigma-Aldrich) solution for 3 h. Subsequently, the devices were cleaned with pure ethanol for a few seconds to prevent the excessive doping effect. For the p-type semiconductor, a DNTT (Sigma-Aldrich) layer was deposited to form a heterojunction structure using thermal evaporation at 25 °C. Patterning was performed using a shadow mask. The thicknesses of DNTT varied from 5 to 50 nm. Then, 70 nm thermally evaporated Au electrodes were deposited using the shadow mask process. While fabricating the heterojunction devices, ZnO and DNTT single-channel devices were also fabricated as references. Electrical characterization was performed using a semiconductor parameter analyzer (Keithley 4200) at 25 °C in ambient air.

### Device and Logic Circuit Modeling

Device modeling and circuit synthesis were performed using HSpice (Synopsys). A semiphysical analytical TFT model was used to model the device characteristics of the ZnO–DNTT AAS device.^53^ Transient response, power dissipation, and delay of ternary circuits were extracted using the developed ZnO–DNTT AAS device model and compared with the modeling results of the ternary circuits designed with the CNTFET model^50^ and the predictive technology model for 90 nm CMOS.^51^

## Supporting Information Available

Fabrication process flow of the ZnO–DNTT AAS device, XPS analysis of ZnO and DNTT, transfer curves and transconductance of ZnO–DNTT AAS device dependence on voltage bias, transfer curves of ZnO and DNTT single TFT dependence on channel thickness and doping type, various STI schemes, comparison of STI operation, various ternary circuit designs and their operation (Figures S1–S9) and PM ternary device switching table (Table S1) (PDF)
