# Supplementary material for: Demonstration of Anti-ambipolar Switch and Its Applications for Extremely Low Power Ternary Logic Circuits
Source: ACS Nano. 2022 Jun 28;16(7):10994–1003. doi: 10.1021/acsnano.2c03523 (PMC9331138; doi:10.1021/acsnano.2c03523)
Supplement: Supplementary file 1 — nn2c03523_si_001.pdf [file nn2c03523_si_001.pdf]

Supporting Information

Demonstration of anti-ambipolar switch and its applications for extremely low power ternary logic circuits

Yongsu Lee, Sunmean Kim, Ho-In Lee, Seung-Mo Kim, So-Young Kim, Kiyung Kim, Heejin Kwon, Hae-Won Lee, Hyeon Jun Hwang, Seokhyeong Kang and Byoung Hun Lee**

Center for Semiconductor Technology Convergence, Department of Electrical Engineering, Pohang University of Science and Technology, Cheongam-ro 77, Nam-gu, Pohang, Gyeongbuk 37673, Republic of Korea

[*]Corresponding author. E-mail: shkang@postech.ac.kr, bhlee1@postech.ac.kr

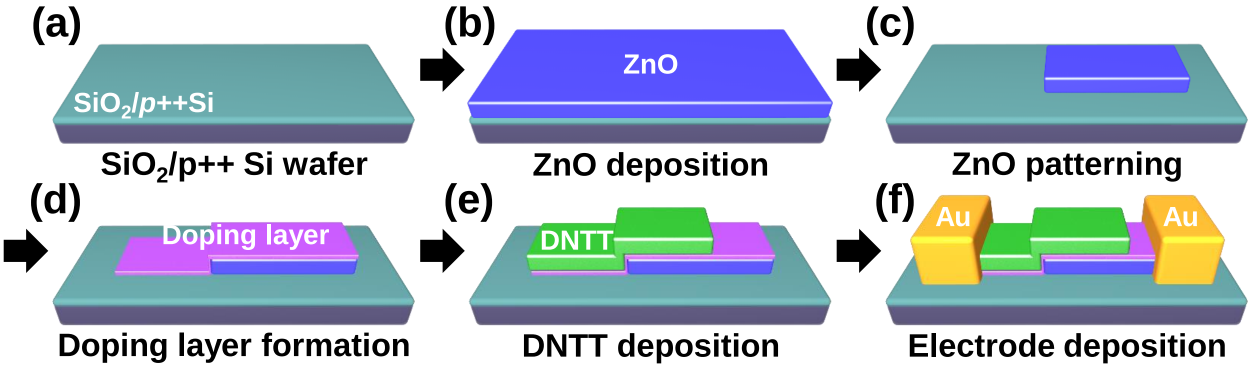

Figure S1. (a–f) Schematic diagrams of the fabrication process flow of the ZnO–DNTT AAS device.

Figure S1 shows the schematic diagrams of the fabrication process flow of the ZnO–DNTT AAS device. Firstly, 90-nm SiO_2/*p*++ Si substrates (used as a back gate) were cleaned with acetone, isopropyl alcohol, and distilled water (DI) for 5 mins in sequence with sonication (Figure S1(a)). Then, ZnO layers were deposited using the atomic layer deposition (ALD) process at 120 °C using diethyl zinc (DEZ) precursor and H_2O oxidant (Figure S1(b)). The thicknesses of ZnO varied from 19 to 50 nm by controlling the cycle of the ALD process. Then, the ZnO layers were patterned using photolithography and a wet etch process using a 1 % HCl solution diluted by DI (Figure S1(c)). To perform chemical doping, the surface of active ZnO was submerged into 0.2 wt% ethanol-diluted branched polyethylenimine (PEI, Sigma Aldrich) or poly(acrylic acid) (PAA, Sigma Aldrich) solution for 3 h. After that, the devices were cleaned with pure ethanol for a few seconds to prevent an excessive doping effect (Figure S1(d)). For the *p*-type semiconductor, a DNTT (Sigma Aldrich) layer was deposited to form a heterojunction structure using thermal evaporation (Figure S1(e)). Patterning was performed using a shadow mask. The thicknesses of DNTT varied from 5 to 50 nm. Then, 70-nm thermally evaporated Au electrodes were deposited with a shadow mask process (Figure S1(f)).

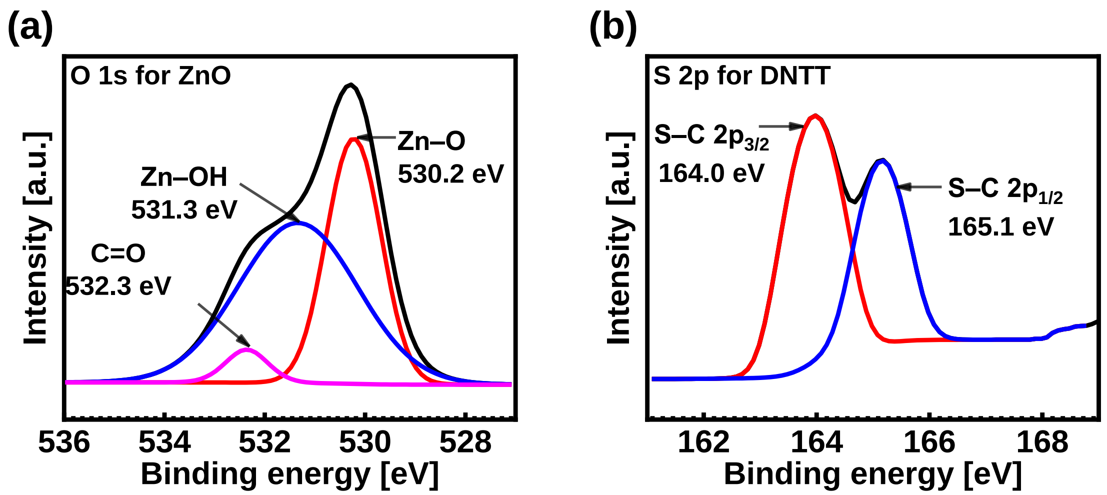

Figure S2. The XPS analysis of semiconductors for (a) ZnO O 1s and (b) DNTT S 2p.

Figure S2 shows the XPS spectra of (a) ZnO O 1s and (b) DNTT S 2p, respectively. For ZnO, the high intensity of Zn–O peak indicates that the crystal structure of the ZnO channel is the wurtzite structure. Zn–OH peak is induced by the OH group at the surface of ZnO, and C=O peak is induced by the carbon contaminations from less disassembled DEZ precursor. As indicated by the TEM cross-section (Figure 1(c)), the structure of the ZnO layer deposited using ALD is similar to the bundles of nanorods[S1]. For DNTT, sulfur atoms are conjugated with carbon atoms by single and double bonding, which is consistent with the XPS result of reference[S2].

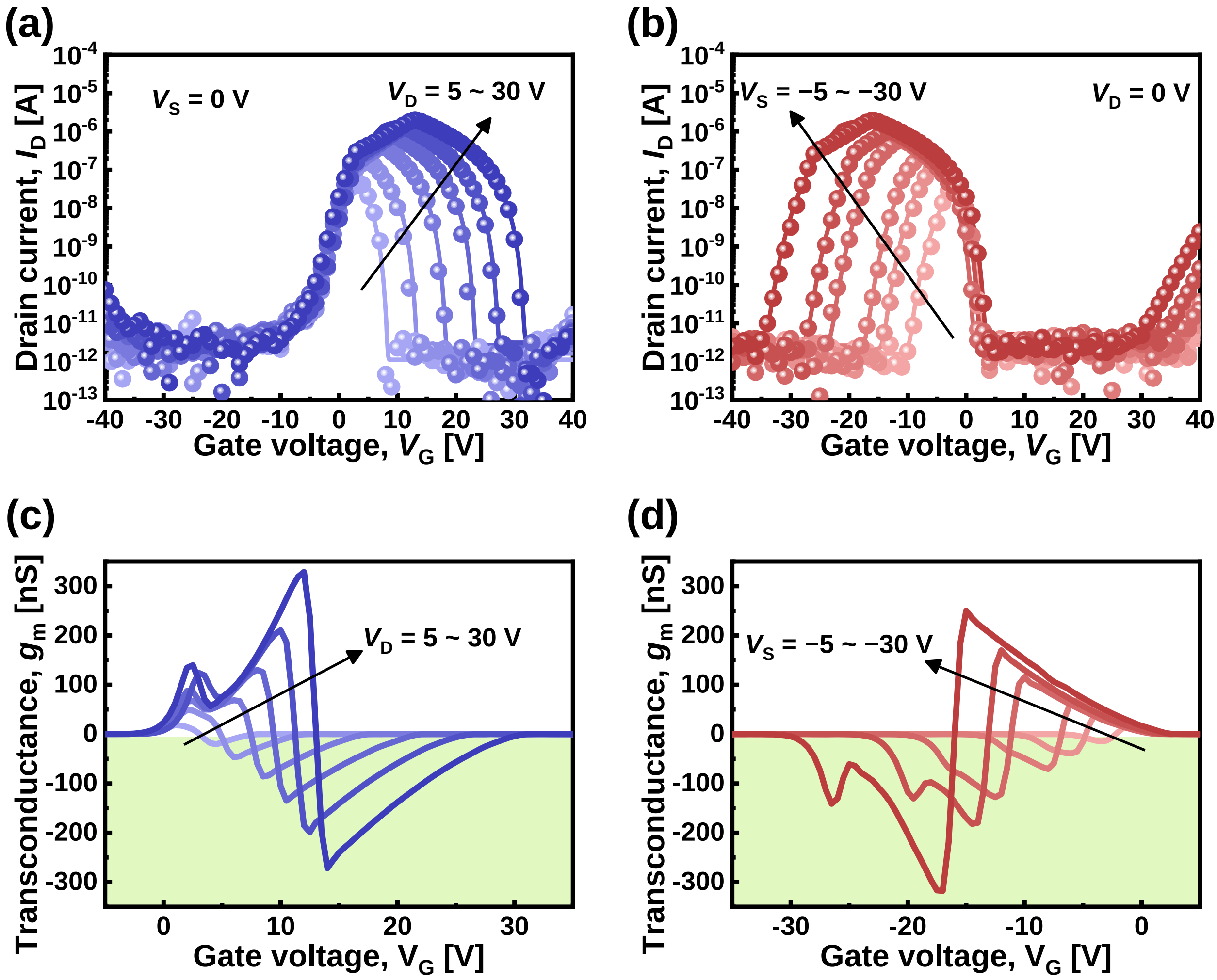

Figure S3. I_D–V_G curves of ZnO–DNTT AAS device using 29 nm thick ZnO and 50 nm thick DNTT with (a) V_D from 5 to 30 V and (b) V_S from −5 to −30 V. Symbols indicate experimental data and lines indicate device modeling fitting results. Negative g_m property of the AAS device with (c) V_D from 5 to 30 V and (d) V_S from −5 to −30 V.

Figure S3(a–b) show drain bias (V_D) and source bias (V_S) dependence on the electrical characteristics of the AAS device, respectively. As V_D is biased on the DNTT side, $V_{th,p}$ of the AAS device varied because V_D affects the number of accumulated hole carriers at the *p*-type channel. Likewise, $V_{th,n}$ of the AAS device varied by V_S on the ZnO side because the number of accumulated electron carriers at the *n*-type channel changed. As V_D and V_S increased, I_{PEAK} increased from 10^{-7} to 10^{-6}. Figure S3(c–d) is g_m of the AAS device according to V_D and V_S, respectively. Negative g_m values are shown in both cases, which reached −272 nS at V_D = 30 V and −318 nS at V_S = −30 V.

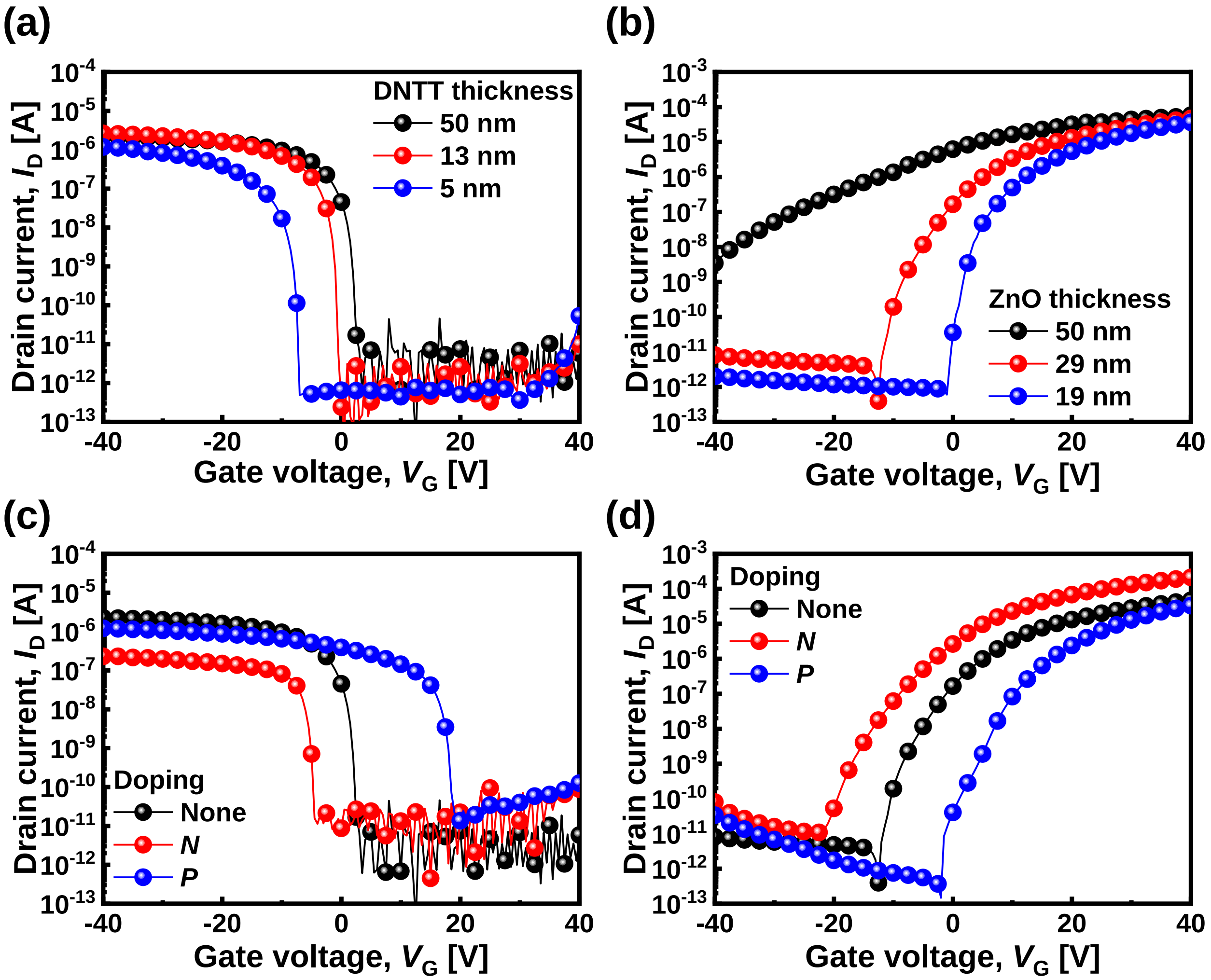

Figure S4. I_D–V_G curves of (a) DNTT and (b) ZnO single layer TFTs with different channel thicknesses at $V_D = \mp 5$ V. I_D–V_G curves of (c) 50 nm DNTT and (d) 29 nm ZnO single layer TFTs before and after the doping at $V_D = \mp 5$ V.

Figure S4(a) shows DNTT TFT characteristics with various DNTT channel thicknesses. As the DNTT channel thickness increases, the hole charge concentration tends to increase, so V_{th} of the DNTT TFT shifts to positive V_G[48]. The V_{th} changes from −7.5 to 2 V as DNTT channel thickness increases from 5 to 50 nm. Figure S4(b) shows ZnO TFT characteristics depending on ZnO channel thickness. The electron charge concentration of the ZnO channel increases as the ZnO channel thickness increases in the same way[25,47]. The V_{th} of ZnO TFT changes from 2 to −40 V as ZnO channel thickness increases from 19 to 50 nm.

Figure S4(c–d) show chemical doping effects on DNTT and ZnO TFTs, respectively. Chemical doping layers of PEI or PAA were placed under 50 nm of DNTT and 29 nm of ZnO channels. PEI induces electron carriers on the channel, so V_{th} of both DNTT and ZnO TFTs shifts

toward the negative V_G. On the other hand, PAA induces hole carriers on the channel, so V_{th} the TFTs shift toward the positive V_G. For DNTT TFT, V_{th} shifts from 2 V to −5 and 18 V by PEI and PAA, respectively. Compared to Figure S4(a), V_{th} shift by chemical doping is more effective than V_{th} shift by changing DNTT channel thickness. For ZnO TFT, V_{th} shifts from −8.5 V to −17 and 4.5 V by PEI and PAA, respectively.

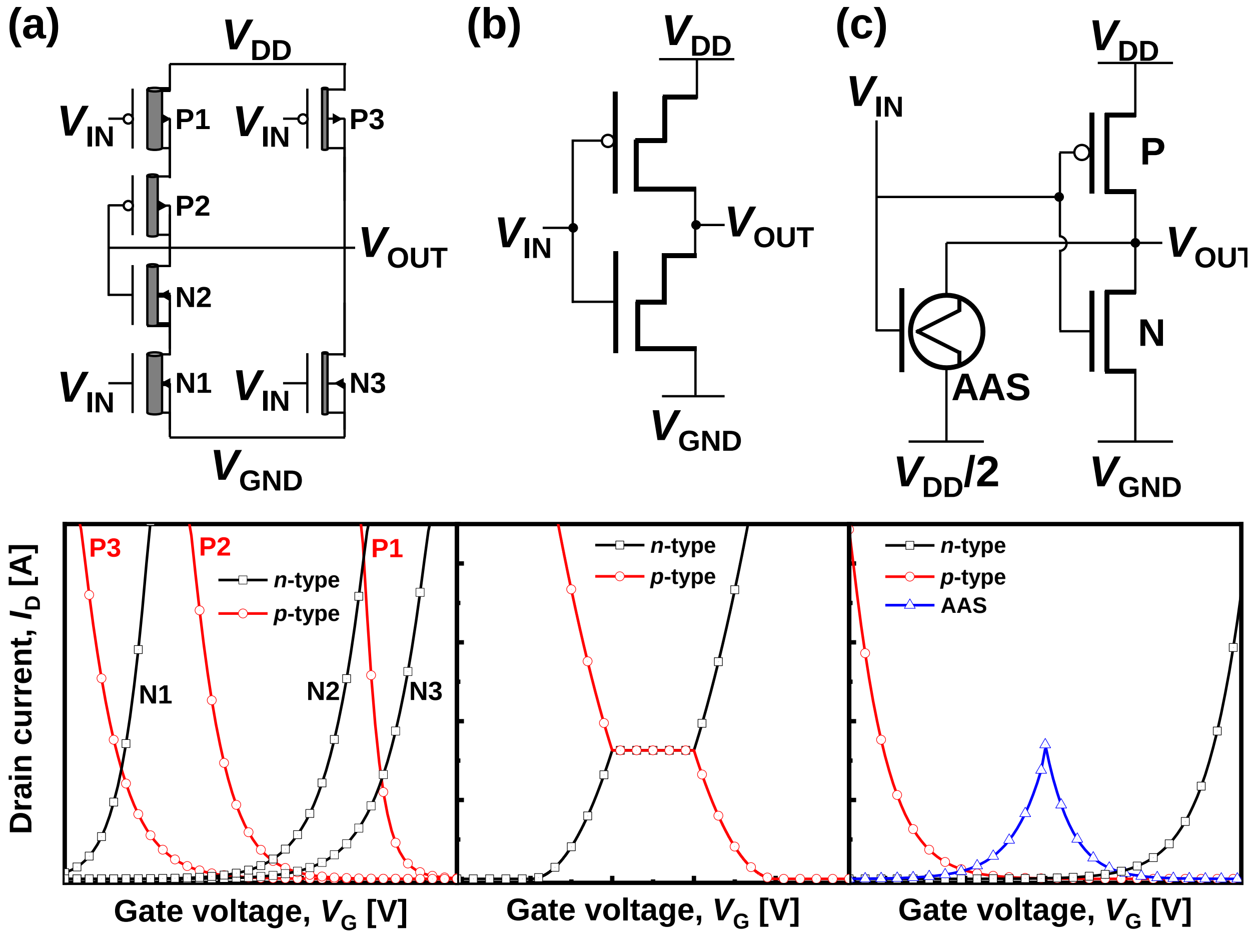

Figure S5. Various STI types as (a) CNTFET-based multi-V_{th} scheme[18,19,21], (b) stack-channel ternary device-based scheme[22–25], and (c) AAS device-based SPTT scheme.

Figure S5 shows various STI types with different ternary circuit schemes. Figure S5(a) shows STI using multi-V_{th} binary CNTFETs. Four devices are connected in parallel as PU and PM network, and two devices (P2, N2) are connected to generate half V_{DD} using negative feedback. This approach has the advantage of strong noise immunity due to the negative feedback elements, but the number of devices considerably increases, in which STI needs six devices. Figure 5(b) shows the STI using devices having an intermediate state. The STI needs only two devices, but the stable ternary device should be demonstrated. Moreover, both above ternary circuit schemes have the static power dissipation problem at the intermediate state. Figure S5(c) shows AAS device-based SPTT STI. In this circuit, PM network is connected additional power rail for $V_{DD}/2$ to represent state 1, so it prevents the intrinsic leakage current at the intermediate. Furthermore, the AAS device can reduce the device count required in PM network, so only three devices are needed to design the STI.

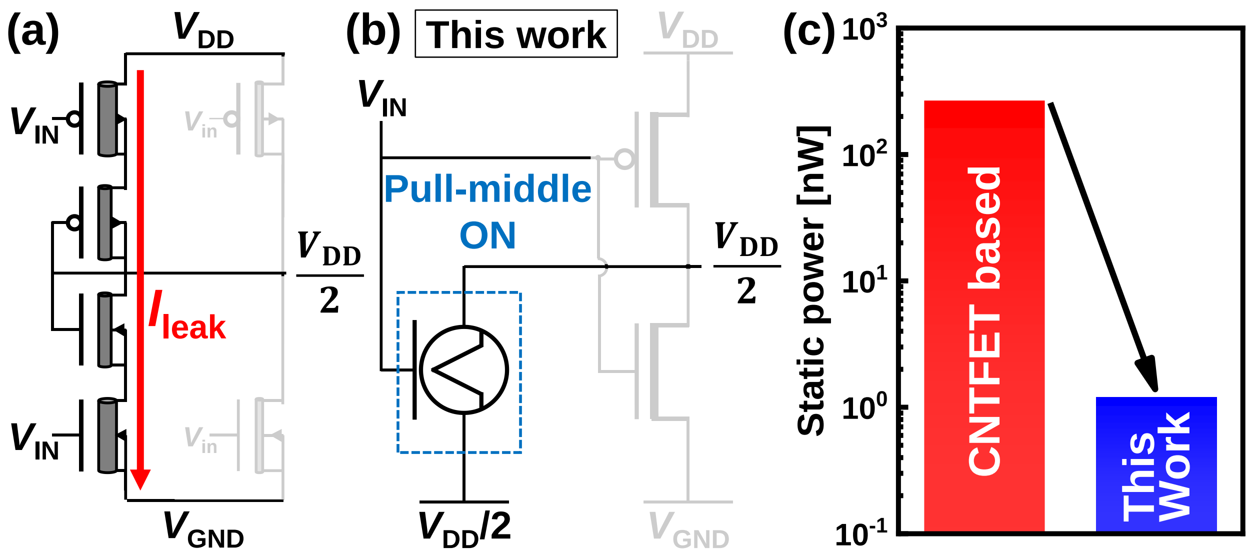

Figure S6. STI operations for (a) CNTFET-based circuit and (b) AAS-based SPTT circuit, and (c) comparison of static power dissipation of both circuits.

The static power dissipation problem from intrinsic leakage in previous ternary schemes is shown in Figure S6(a). When $V_{OUT} = V_{DD}/2$, PU and PD networks are half-turned on. It generates a leakage current path between V_{DD} and V_{GND} power rails. The leakage currents are of μA to nA order[19,25]. On the other hand, the STI with an AAS device has a relatively small leakage current because $V_{DD}/2$ is just transited only from PM network (Figure S6(b)). The leakage current is of pA level, which is off current levels of devices. Overall, the static power of an STI with an AAS device can be reduced to a sub-nW level, which is ~1/100 the typical value of a CNTFET-based ternary STI (Figure S6(c)).

Table S1. PM ternary device switching table.

Pull-middle ternary devices					
Pull-middle Network	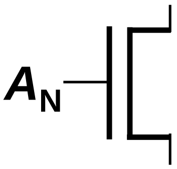	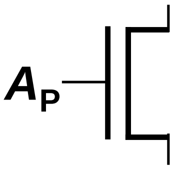	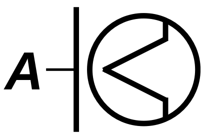	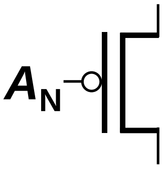	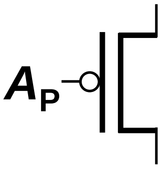
Switching operation					
Input = 0	ON	ON	OFF	OFF	OFF
Input = 1	OFF	ON	ON	ON	OFF
Input = 2	OFF	OFF	OFF	ON	ON

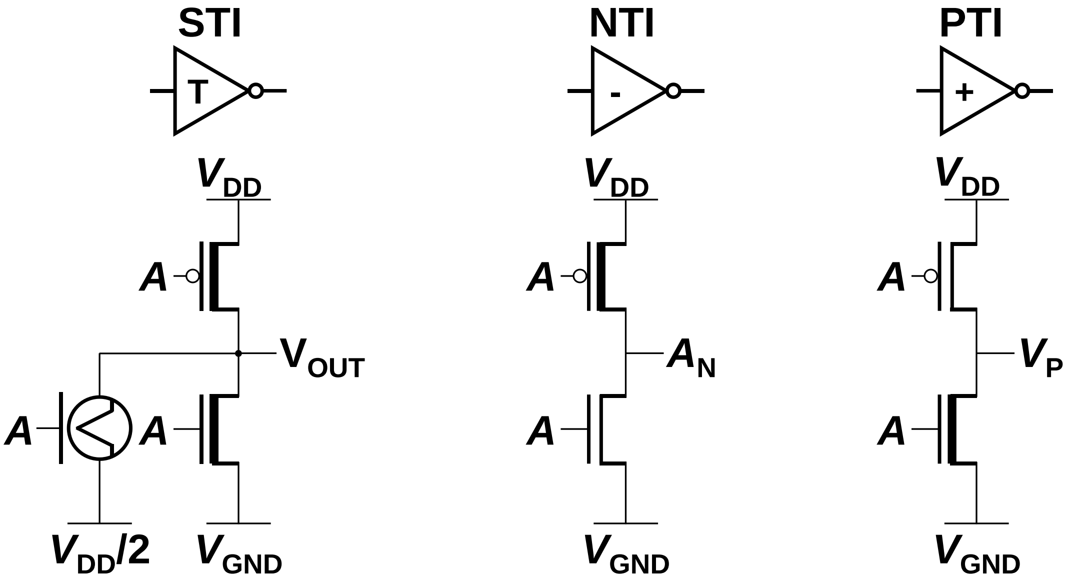

Figure S7. Schematic diagrams of the proposed various transistor-level of STI, NTI, and PTI.

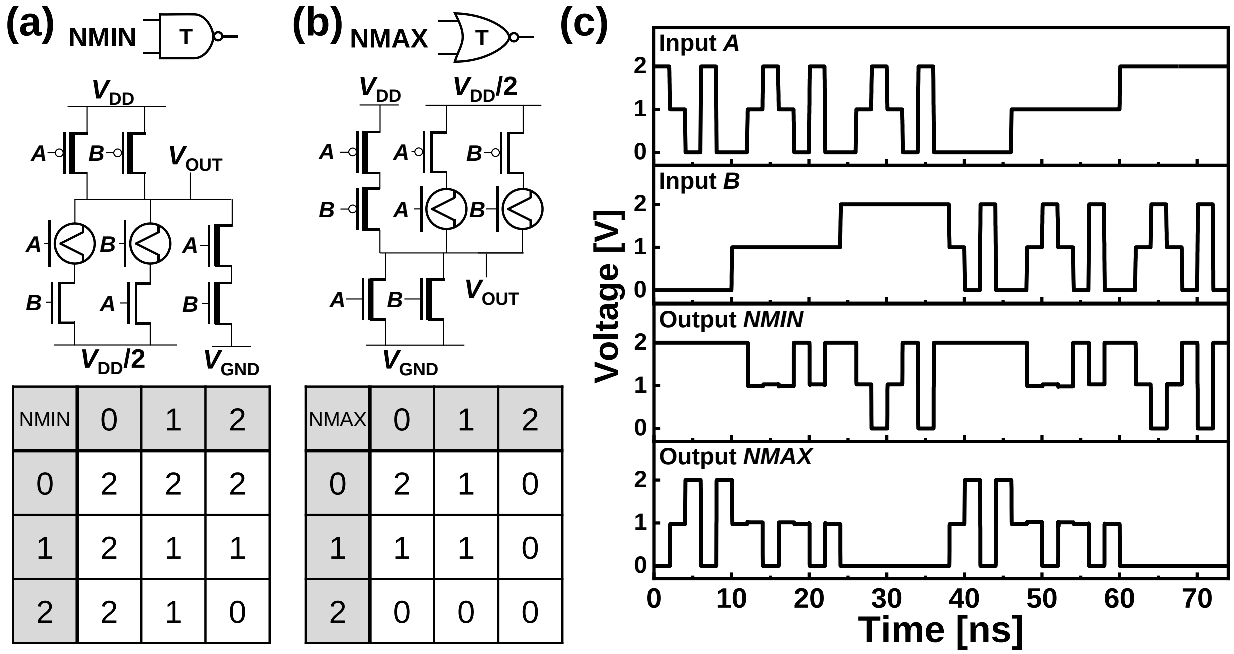

NMIN	0	1	2
0	2	2	2
1	2	1	1
2	2	1	0

NMAX	0	1	2
0	2	1	0
1	1	1	0
2	0	0	0

Figure S8. Schematic diagrams of the proposed various transistor-level of ternary logic circuits and truth tables for (a) NMIN and (b) NMAX. (c) transient responses of the NMIN and NMAX.

Figure S8(a–b) show circuit designs and truth tables of NMIN and NMAX using the AAS device-based SPTT scheme. Eight devices are needed to design each circuit, whereas 10 devices are needed for the CNTFET-based multi-V_{th} scheme. Figure S8(c) shows the transient responses of proposed NMIN and NMAX at 250 MHz frequency. Compared to the truth table in Figure S8(a–b), all logic functionalities are successfully confirmed.

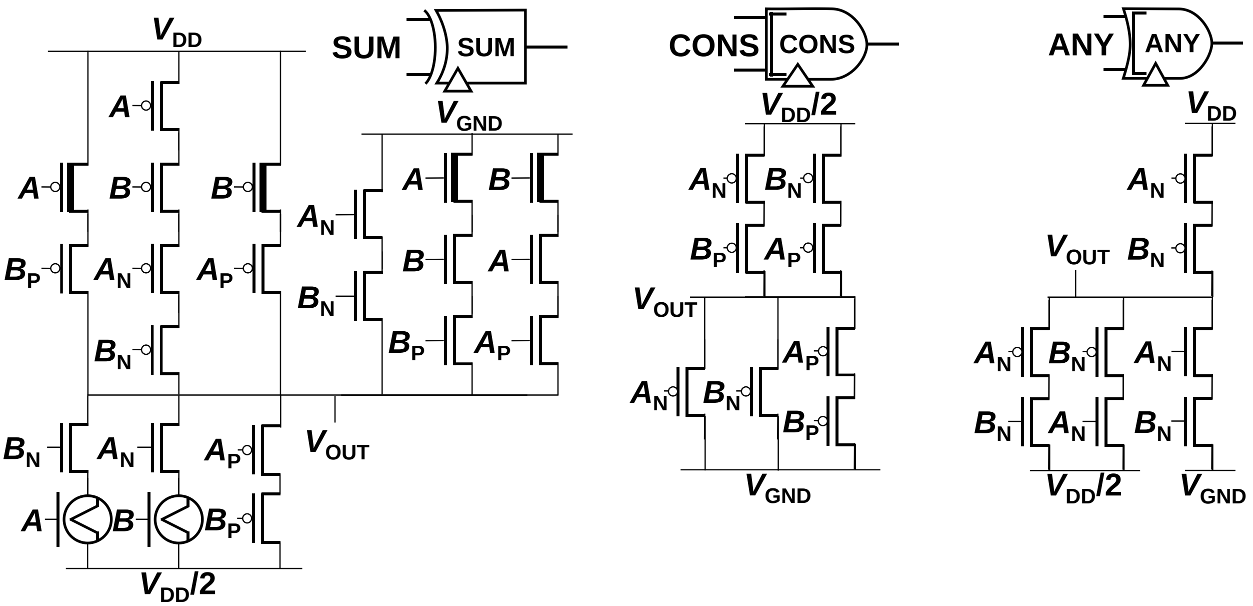

Figure S9. Schematic diagrams of the proposed various transistor-level of SUM, CONS, and ANY.

REFERENCES

(S1) Al-Gaashani, R.; Radiman, S.; Daud, A. R.; Tabet, N.; Al-Douri, Y. XPS and Optical Studies of Different Morphologies of ZnO Nanostructures Prepared by Microwave Methods. *Ceramics International* **2013**, *39* (3), 2283–2292. https://doi.org/10.1016/j.ceramint.2012.08.075.

(S2) Yue, H.; Kong, L.; Wang, B.; Yuan, Q.; Zhang, Y.; Du, H.; Dong, Y.; Zhao, J. Synthesis and Characterization of Novel D-A Type Neutral Blue Electrochromic Polymers Containing Pyrrole[3-c]Pyrrole-1,4-Diketone as the Acceptor Units and the Aromatics Donor Units with Different Planar Structures. *Polymers* **2019**, *11* (12), 2023. https://doi.org/10.3390/polym11122023.
